# Collaborative model of care between Orthopaedics and allied healthcare professionals trial (CONNACT) – a feasibility study in patients with knee osteoarthritis using a mixed method approach

**DOI:** 10.1186/s12891-020-03611-9

**Published:** 2020-09-04

**Authors:** Bryan Yijia Tan, Benjamin Tze Keong Ding, Michelle Jessica Pereira, Soren Thorgaard Skou, Julian Thumboo, Josip Car

**Affiliations:** 1 Department of Orthopaedic Surgery, Woodlands Health Campus, National Healthcare Group, Singapore, Singapore; 2 Lee Kong Chian School of Medicine, Nanyang Technological University, Singapore, Singapore; 3 Ministry of Health Holdings, Singapore, Singapore; 4 Health Services Outcome Research, National Healthcare Group, Singapore, Singapore; 5 Research Unit for Musculoskeletal Function and Physiotherapy, Department of Sports Science and Clinical Biomechanics, University of Southern Denmark, Odense M, Denmark; 6 Department of Physiotherapy and Occupational Therapy, Næstved-Slagelse-Ringsted Hospitals, Slagelse, Denmark; 7 Singapore General Hospital, Singapore, Singapore

**Keywords:** Knee, Osteoarthritis, Model of care, Pilot, Feasibility. Mixed methods

## Abstract

**Background:**

Osteoarthritis is a leading cause of global disability resulting in significant morbidity and cost to the healthcare system. Current guidelines recommend lifestyle changes such exercises and weight loss as first line treatment prior to surgical consideration. Our current model of care is inefficient with suboptimal allied health intervention for effective behaviour changes. A 12-week community based, individualized, multidisciplinary new model of care for knee osteoarthritis was developed in light of current deficiencies.

**Methods:**

The primary aim of this study was to determine the feasibility of a full randomized controlled trial evaluating this new model of care using pre-defined progression criteria. The secondary aim was to optimize the intervention and study design through a process evaluation. A pilot exploratory, parallel arm, single blinded randomized trial design using a mixed method approach was utilized. Progression criteria for a full trial including key domains of patient recruitment and retention, outcome measure acceptability and improvement, adverse events were developed. The primary outcome measure was the Knee Injury and Osteoarthritis Outcome Score (KOOS) at baseline and 12-weeks. Secondary outcomes included quality of life, functional and psychological assessments. Semi-structured interviews were conducted with the patients at 12-weeks.

**Results:**

20 patients (3 males, 17 females) were randomized (10 intervention, 10 control). Intervention arm patients reported better improvements in their knee function, quality of life, psychological outcome, dietary improvement and weight loss compared to the control arm at 12-weeks. Semi-structured interviews revealed several themes pertaining to feasibility and intervention optimization. 5 out of the 6 progression criteria’s domains were met (recruitment criteria not met).

**Conclusion:**

This pilot has demonstrated the feasibility of a full randomized control trial investigating the potential effectiveness of the new proposed model of care for knee osteoarthritis using pre-defined progression criteria and process evaluation. Results from the qualitative study were used to modify and improve the intervention content, delivery model and study design for a large effectiveness-implementation hybrid randomized control trial that is currently underway.

**Trial registration:**

Retrospectively registered on 18 January 2019 at http://clinicaltrial.gov ID: NCT03809975.

## Background

With a rapidly aging population, Musculoskeletal (MSK) disorders account for the largest cause of disability around the world. In particular, osteoarthritis (OA) is currently the 11th highest global cause of disability [1]. International guidelines are consistent in their recommendations for individualized lifestyle changes, especially exercise and weight loss programs to manage knee OA and recommend a stepwise approach where surgery is considered when non-surgical treatment fails [2, 3]. Yet, international studies report that at least 60% of patients from established healthcare systems around the world such as Australia, Canada and the US are not receiving optimal non-surgical treatment [4–6].

There remains suboptimal use of non-surgical treatment such as allied health interventions (e.g. delivered by physiotherapist and psychologists) to support effective lifestyle and behaviour changes in most models of care [7]. As a result, surgery is at present often a result not from a failure of non-surgical treatment but failure of the healthcare system to provide adequate and efficient conservative treatment. The literature suggests that at least a quarter of knee arthroplasty could have been avoided through optimal non-surgical treatment^8^. In the United States, it is anticipated that knee arthroplasty rates will rise by 673% between 2005 to 2030 [8]. In Australia similarly, it is anticipated there will be a 276% increase between 2013 to 2030 [9]. While knee arthroplasty surgery has been shown to be an effective option for knee OA, it is not without risks, complications or downsides. Firstly, it is expensive [10]. Secondly, complications while uncommon can still occur and certain complications such a popliteal artery damage or deep infection can have devastating consequences [11]. Thirdly, as knee arthroplasty have a limited lifespan, the earlier surgery is done the higher the likelihood of a revision surgery being required in the future [12]. Fourthly, up to 25% of patients remain unsatisfied [13] and up to 34% of patients have unfavourable long-term pain outcomes post-surgery [14].

There is an urgent need for new Models of Care (MoC) for OA by optimizing evidence-based non-surgical treatments to deliver value-based care. Combination treatments have shown promise in literature with several trials demonstrating effectiveness mostly in exercise and nutritional combination interventions [15, 16]. More recent studies have looked at the effectiveness of other combinations including self-management education programs and psychological support [17, 18]. Very few studies have looked at incorporating multiple components in a complex intervention [19]. The **Collaborative Model of Care between Orthopaedics and Allied Healthcare Professionals (CONNACT)** model of care is a complex intervention that was developed in to meet this gap in literature. CONNACT is a community-based, multidisciplinary (Orthopaedics, Physiotherapy, Dietetics, Psychology and Social Work) 12-week program that uniquely uses an individualized approach based on a triaging criterion to tailor the treatment to each patient in line with the “right care, right time, right team, right place” philosophy of any successful model of care.

## Methodology

### Aim and study design

The primary aim of this study was to determine the feasibility of a full randomized controlled trial (RCT) using pre-defined progression criteria. The secondary aim was to optimize the intervention and study design through a process evaluation in preparation for a full RCT. The RCT will test the clinical effectiveness of the community-based, individualized, multidisciplinary model of care for knee OA (CONNACT) compared to usual care.

A pilot exploratory, parallel arm, single blinded randomized trial design was used for the proof of concept, feasibility study based on the conceptual framework developed by Eldridge et al. [20]. Guidelines based on the OA Research Society International (OARSI) clinical trials recommendations were used in the design of the study [21]. It was conducted as a single centre pragmatic pilot randomized trial. Ethics approval was obtained through the Institution Review Board prior to the conduct of the study. The Consolidated Standards of Reporting Trials (CONSORT) checklist for pilot studies was used to ensure comprehensive reporting of this pilot study [22].

### Participants and recruitment process

Patients who were referred by a primary healthcare or emergency medicine doctor to the Outpatient clinic at the Department of Orthopaedic Surgery at Tan Tock Seng Hospital, a tertiary referral centre in Singapore with a suspected diagnosis of knee OA were screened based on the inclusion and exclusion criteria presented in Table 1. All referral letters were screened based on electronic medical records for inclusion and exclusion criteria. Patients who were eligible were invited to attend a recruitment clinic where they were assessed by the study team and invited to participate in the study if they met all the inclusion and exclusion criteria. Recruitment clinics were carried out in the Tan Tock Seng Hospital Specialist Outpatient Clinic.

**Table 1:** Inclusion and Exclusion Criteria

Inclusion Criteria (all 4 must be present)	Exclusion Criteria
National Institute of Health and Care Excellence (NICE) clinical criteria for knee OA [23] 1. Age ≥ 45 years old **and** 2. Has activity related knee pain **and** 3. Has either no morning knee-related stiffness or morning stiffness than last no longer than 30 min	Alternative diagnosis to knee OA e.g. Referred pain from the spine or hip
Radiographic severity of knee OA, Kellgren-Lawrence Score [24] > 1	Secondary arthritis e.g. inflammatory, post-traumatic
Knee Injury and OA Outcome Score [25] (KOOS_4_) ≤ 75	Inability to comply with study protocol e.g. cognitive impairment
Community ambulator with or without walking aid	Previous knee arthroplasty
	Wheelchair bound patients
	Medical condition that will medically interfere with study involvement e.g. decompensated heart failure, stroke, end stage renal failure

The choice of inclusion and exclusion criterion were based on similar studies [19] with the primary intent of identifying patients with primary knee OA who were sufficiently disabled from the condition both from functional and radiological perspective but would likely benefit from an intervention (medical fitness, ability to follow study protocol and community ambulator).

### Intervention – CONNACT model of care

A MoC is an evidence informed policy or framework that outlines the optimal manner in which a condition should be managed by addressing the principles of care and how it should be implemented in the local setting. “*Right care*, delivered at the *right time*, by the *right team*, in the *right place*, with the *right resources*” is the ideal end state for any MoC [26].

The CONNACT MoC was developed starting with a throughout literature search on the best practices in knee osteoarthritis care [3, 27], a review of successful programs [7], international collaborations with the Good Life with Osteoarthritis in Denmark (GLA:D) program from Denmark [28] and Osteoarthritis Chronic Care Program (OACCP) from Australia [29]. Collaborators from the GLA:D and OACCP shared best practices from their programs and gave expert advice on program development. Adapting it to our local context was done through engagement with local experts from the different speciality groups.

The CONNACT MoC principles are highlighted below based on the key goals of any MoC.

1. **Right Care** - Fundamental paradigm shift by moving from an acute episodic type treatment generally associated with OA to a chronic disease model of care [30] with a focus on patient empowerment, behavioural modification in a multidisciplinary team community based approach grounded in best practices. The intervention consisted for 4 main components.
  - Clinical Assessment and Education
  - Exercise therapy
  - Nutrition and Dietetics
  - Psychological Support
  Each component was designed to enhance the other components synergistically e.g. behavioural change strategies taught during the psychological class is used to improve compliance to exercise and diet modification.
2. **Right Time** – Individualized care for each patient based on a triaging criterion e.g. nutrition class for obese patients to ensure timely and appropriate care for patients
3. **Right Team** – Multidisciplinary team consisting of Orthopaedic surgeons, Physiotherapists, Dieticians, Psychologists, Social workers
4. **Right Place** – Community based to bringing care closer to the patients and remove the disease stigma associated with a hospital environment. The intervention was conducted at Ang Mo Kio St Luke Eldercare Centre, a community-based rehabilitation centre with exercise facilities.

Triaging Criterion were developed to individualize treatment from the onset. All patients would receive the education and physical exercise components. A BMI cut off was used as a criterion for dietician intervention. In light of the significant impact of psychological conditions (anxiety, depression) and pain intensity and interference in predicting outcomes in osteoarthritis patients [31, 32], Patient Health Questionnaire-4 (PHQ-4) [33] a 4-item screening assessment for depression and anxiety and Pain Intensity, Enjoyment of life and **G**eneral Activity (PEG) [34], an 3-item assessment of pain intensity interference derived from the Brief Pain Inventory (BPI) [35] was selected as appropriate triaging tools in addition to outcome measures.

Intervention will be delivered in a group based format as group-based interventions have been shown to be more effective compared to individual interventions in promoting physical activity through cohesion and peer support [36]. Numbers for the class were kept between 8 and 10 participants per class to ensure good participant facilitator ratio and optimize group dynamics.

Details for each intervention component including the triaging criteria, healthcare professional delivering the intervention and treatment principles are summarized in Table 2.

**Table 2:** Intervention Summary

Intervention Component	Criteria to receive intervention	Healthcare Professional	Treatment Principles	Delivery
Exercise Therapy	All patients	Physiotherapist	American College of Sports Medicine (ACSM) [37] Neuromuscular Exercise (NEMEX) [28] guidelines	Group sessions × 6
Clinical Assessment and Education	All patients	Orthopaedic Surgeon	Clinical and Radiological Assessment Pharmacological Intervention	Group Education sessions × 2
Dietetics and Nutrition	BMI > 23.5^a^	Dietician	Dietary intervention to increase dietary related nutrition knowledge and self-efficacy for effective weight loss [38]	Group sessions × 3
Psychological support	Patient Health Questionnaire 4 (PHQ-4) > 5 or Pain, Enjoyment, General Activity Scale (PEG) > 4 on all scales	Psychologist Medical Social Worker	Acceptance and Commitment Therapy (ACT) principles [39, 40] Pain Management Coping Strategies Improving compliance to behavioural modifications	Group sessions × 3

^a^ A lower cut off of BMI 23.5 is recommended for Asians [41]

### Usual care

Usual care constituted a referral to the outpatient physiotherapist at the tertiary hospital where patients were seen 1–2 weeks post referral. The physiotherapist would conduct an assessment and recommend a variety of lifestyle modifications and exercise therapy. The type of exercises and number of physiotherapy sessions were at the discretion of the patient and the physiotherapist.

### Outcomes measures

The primary outcome measure used was the Knee Injury and Osteoarthritis Outcome Score (KOOS_4_). KOOS contains 5 domains of questions namely symptoms, pain, function (daily living), function (sports, recreational activities) and quality of life. Consistent with other studies with similar population of elderly patients with knee OA, the function (sports, recreational activities) subscale were deemed to be less relevant for this population and the remaining 4 domains were combined to form a composite score [19]. The KOOS score has been validated in Singapore [42]. Secondary outcomes included KOOS individual subscales, quality of life scoring, functional assessment, diet and psychological related outcomes. Outcome measures were collected at baseline and 12-weeks. Any adverse events were collected through the study during all follow up visits including data collection and intervention visits. Table 3 summarizes all the outcome measures. Baseline data on the patient demographics, socioeconomical status, Co-morbidities and functional status (Charlson comorbidity index [43], Barthel Index for Activities of Daily Living [44]) were collected as well.

**Table 3:** Outcome Measures

Outcome Measure	Variables
Baseline Measures	Demographics (Age, Gender) Socioeconomical status Medical Co-morbidities Functional status (Charlson comorbidity index [43], Barthel Index for Activities of Daily Living [44])
Primary Outcome Measures	Knee Injury and Osteoarthritis Outcome Score (KOOS_4_) [25]
Secondary Outcome Measures	Knee Injury and Osteoarthritis Outcome (KOOS) subscales (pain, symptoms, function, quality of life) Quality of Life EQ-5D-5L [45] Functional Assessment (30s chair stand, 10 m fast paced walk, stair climb, timed up-and-go) Body Mass Index (BMI) Modified Semi-Quantitative Food Frequency Questionnaire (FFQ) [46] Patient Health Questionnaire 4 (PHQ-4) [33] Pain, Enjoyment, General Activity Scale (PEG) [34] Acceptance and Action Questionnaire 2 (AAQ-II) [47] Chronic Pain Acceptance Questionnaire 8 (CPAQ-8) [48] Global Impression of Change (GIC) [49] Adverse events

The EQ-5D-5L value set that has been validated for the Singapore population using a time trade-off method was used to calculate utility values [50]. The choice of functional assessments was based on the recommended OARSI performance test for functional testing in OA [51]. Modification to the original Food Frequency Questionnaire (FFQ) was performed to reduce the length to reduce questionnaire burden and adapt it based on local dietary practices. Scoring was developed based on the weightage of fat/sugar/fibre content of the particular food item based on the energy and nutrient composition reported by the Singapore Health Promotion Board (http://focus.hpb.gov.sg/eservices/ENCF/). Modified FFQ was only done for patients who had a BMI > 23.5.

### Progression criteria to decide whether to proceed with RCT

Randomized Controlled Trials (RCT) are expensive, time consuming endeavours. Having robust progression criteria to a larger, definitive RCT based on pilot data is crucial to objectively determining if the pilot RCT should be developed into a larger, definitive RCT. Based on the guidelines and key considerations proposed by Avery and colleagues for developing and using progression criteria for internal pilot studies [52], progression criteria were developed by the study team (Table 4). The guide highlighted the elements of any pilot study that should be critically evaluated including patient recruitment, intervention adherence, adverse outcome rates and outcome assessment burden. In addition, these progression criteria were recommended to be pre-determined to ensure critical evaluation in establishing RCT viability.

**Table 4:** Progression Criteria for RCT

Domain	Proceed with RCT	Proceed, but changes to the protocol need to be discussed	Do not proceed with main trial unless the problem can be solved
Recruitment	Recruitment of 30 participants with OA within 3 months	Recruitment of 30 participants with OA within 3–6 months	30 participants with OA are not recruited within 6 months
Retention	At least 75% retention of participants through follow up	At least 50% retention of participants through follow up	Less than 50% retention of participants through follow up
Completion of Intervention	At least 75% complete more than half of the intervention	At least 50% complete more than half of the intervention	Less than 50% complete more than half of the intervention
Outcome Measures Acceptability	At least 80% of participants do not find the outcomes so burdensome that they would not participate in the study again	At least 70% of participants do not find the outcomes so burdensome that they would not participate in the study again	Less than 70% of participants do not find the outcomes so burdensome that they would not participate in the study again
Function and/or Quality of Life Improvement	Improvements in function and/or quality of life found by at least 50% of the participants	Improvements in function and/or quality of life found by at least 25% of the participants	Improvements in function and/or quality of life found by less than 25% of the participants
Adverse events	No serious care-related adverse events during follow up	Less than five serious care-related adverse events during follow up	Five or more serious care-related adverse events during follow up

In assessing patient recruitment, the guide recommended the use of a rates per unit time. Considering the average time for grant funding (2–3 years) and the average sample size (100–150 patients) used by similar studies [19, 53], a target of 30 patients in 3 months was set to ensure that sufficient recruitment would be achievable during the main RCT assuming a consistent recruitment strategy to the pilot study was followed. A low threshold for adverse outcome was set with a target of no serious care-related adverse event. A serious care-related adverse event was defined as an event related to the treatment that was limb or life threatening or resulted in hospitalization. Targets for completeness of intervention (75%), retention (75%) and outcome measures acceptability (80%) were set to ensure that loss of follow-up or missing data would be kept to a minimum for the main trial. Completeness of intervention was defined as finishing more than half the intervention. Retention was the defined as the number of patients who completed the study including the final outcome measure. Outcome measure acceptability was defined as the numbers of patients do not find the outcomes so burdensome that they would not participate in the study again. Function or quality of life improvement targets were set at 50% to ensure intervention effectiveness. Improvement was defined a positive change in either functional or quality of life outcomes.

Avery et al. recommended the use of a red/amber/green traffic light system instead of a simple stop/go basis. An amber light would indicate that a change in protocol is recommended before proceeding with the main RCT while a red light would indicate serious issues with the study and until those issues were resolves, proceeding to an RCT would not be recommended.

### Sample size

Whitehead et al. proposed a method using the standardized effect size to estimate the sample size for a pilot randomized trial instead of using the rule of thumb method which ranged from 24 to 70 patient sample size [54]. Based on an estimated standardized effect size 0.5 reported by a systematic review and meta-regression analysis of RCTs for exercise based interventions for knee OA in pain and disability [55], assuming 90% power and two-sided 5% significance, 15 patients in each arm were recommended.

### Randomization and data collection

Study data were collected and managed using REDCap electronic data capture tools [56, 57]. Patients who consented to participate were randomized (1; 1 allocation ratio) between the intervention and usual care using a permuted block randomization method using block sizes of 4,6 and 8. The random allocation sequence was generated by an independent statistician and was kept concealed from the study team. Randomization was done using the REDCap randomization module and allocation was locked once assigned. Randomization was only performed after the patient was counselled fully about the study, had provided informed consent and baseline data was collected.

### Blinding

Outcome measures were measured by blinded outcome assessors. The outcome assessors received training prior to study initiation to ensure good inter- and intra-observer reliability particularly for the functional outcome testing. Patients were instructed not to reveal their allocation to the outcome assessors.

### Statistical analysis

The results were analysed using an intention-to-treat (ITT) principle. Data was entered and analysed using the IBM SPSS Statistical Software Version 25. The data was checked for completeness and consistency prior to analysis. Descriptive frequency analysis was used for baseline characteristics. For continuous variables, the mean and standard deviation were reported. For categorical variables, the frequencies and percentages were reported. In view of the sample size, independent hypothesis testing using non-parametric Mann Whitney U test to look for differences between intervention and control group. The median and interquartile range were presented with the corresponding *p* values. The significance level was set at 5%.

### Process evaluation

Through purposive sampling, semi-structured interviews were conducted with the intervention arm patients at 12-weeks as part of the process evaluation. The Medical Research Council (MRC) has developed a set of guidelines for the conduct of process evaluations [58]. MRC recommends a basic framework for process evaluation with the emphasis being different at each stage of the study. In the pilot phase, the key is in understanding the feasibility and intervention design optimization. The interview guide and questions (Table 5) were based on the key emphasis on feasibility for a full RCT based on the proposed progression criteria above and intervention design optimization based on the MRC guidance.

**Table 5:** Interview Topic Guide

Topic	Questions
Intervention Design Optimization	Did you feel that you benefitted from the intervention? Why?
	What specific part of the intervention did you find most useful? Why?
	Did you find being in a group helpful or would you have preferred more individual attention?
	Do you have any suggestions on how we can make the program better?
Feasibility	Were you able to complete the whole program? Were there external reasons that prevented your full participations?
	Would you participate again in the program if given a chance? Would you recommend your friends to participate in the program?
	Did you find the outcome measures too burdensome to complete? Which ones?

The semi-structured interviews were conducted by a research assistant, TCY, who was involved with the patient recruitment and coordination of care. The potential bias the interviewer had on the patients as part of the study team was balanced by the fact that having journeyed with the patients, she had gained the trust of all the patients, most of them who were willing to share with her their personal problems. As a member of the study team but yet not a healthcare professional who delivered the intervention, patients were more open to share with her their honest opinions. In addition, having seen the entire 12-week process, she was in an ideal position to probe intelligently during the interview guided by the topic guide. Extensive hand written notes and quotes were noted down during the interview and the results were interpreted through a thematic analysis by a senior researcher (BTY) with qualitative experience.

## Results

### Participants and baseline characteristics

**Fig. 1:**
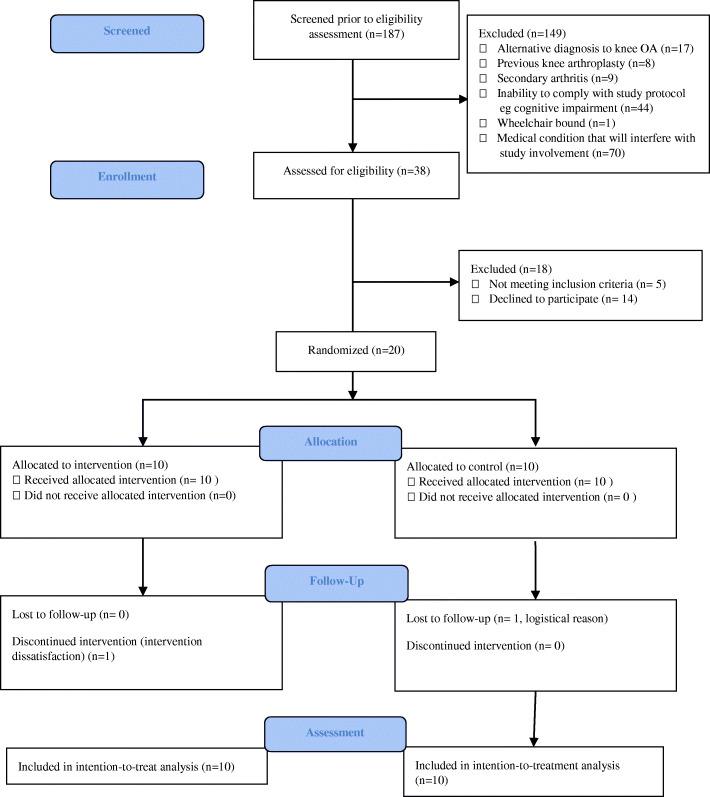
Flow diagram

From late August to early November 2018, over a period of 3 months, a total of 20 patients were recruited (10 control and 10 intervention). Final follow up at 3 months were completed in February 2019. Control group patients were younger with a mean age of 59.6 years old with a distribution of 7 females and 3 males. The intervention arm patients had a mean age of 68.0 years old and was made up of all females. Baseline characteristics are presented in Table 6 and study flow in Fig. 1.

**Table 6:** Baseline Characteristics

	Control (*n* = 10)	Intervention (*n* = 10)
Age (years), mean (SD)	59.6 (6.52)	68.0 (8.11)
Women, n (%)	7 (70%)	10 (100%)
Weight (kg), mean (SD)	68.34 (13.97)	60.15 (8.18)
Affected Knee Joint, n (%)		
Right	4 (40%)	2 (20%)
Left	3 (30%)	4 (40%)
Bilateral	3 (30%)	4 (40%)
Radiographic knee OA severity (Kellgren-Lawrence), n(%)^a^		
Grade 2	5 (50%)	2 (20%)
Grade 3	3 (30%)	7 (70%)
Grade 4	2 (20%)	1 (10%)
Barthel Index, mean (SD)	19.7 (0.48)	19.5 (0.53)
Charlson Comorbidity Score, n (%)		
0	6 (60%)	9 (90%)
1	4 (40%)	1 (10%)
2 or above	0	0
Knee Injury and Osteoarthritis Outcome Score, mean^a^ (SD)		
KOOS_4_	55.15 (10.84)	55.34 (12.88)
KOOS symptoms/stiffness	52.50 (16.76)	51.79 (17.52)
KOOS pain	64.72 (15.33)	58.33 (17.67)
KOOS function (daily living)	68.38 (15.90)	67.50 (15.97)
KOOS quality of life	35.00 (14.49)	43.75 (12.15)
Quality of Life, mean (SD)		
EQ-5D Index	0.45 (0.37)	0.49 (0.27)
EQ-5D VAS	73.50 (20.15)	64.50 (15.71)
Psychology, mean (SD)		
Pain, Enjoyment, General Activity Scale	6.33 (2.26)	5.33 (2.05)
Patient Health Questionnaire 4	2.20 (3.91)	3.60 (3.95)
Functional Assessment, mean (SD)		
Timed 10 m walked test (sec)	5.62 (1.06)	7.00 (1.64)
Time up-and-go test (sec)	10.27 (2.51)	12.83 (2.11)
30s chair stand test (count)	9.30 (3.53)	6.90 (3.51)
4 stairs climb test (sec)	8.39 (4.00)	11.94 (6.20)

^a^for bilateral knee OA, the index/most severe joint was used

### Outcomes

In terms of knee function scores and quality of life, there was a clear trend of the intervention arm patients having a higher KOOS_4_, KOOS symptoms/stiffness, KOOS quality of life, EQ-5D VAS. Psychological outcomes wise, there was a clear trend where the PEG was positively impacted to a greater extent at 12-weeks in the intervention group compared to the control group. This was the case of weight where intervention patients lost weight compared to the control arm where patients gained weight after 12-weeks. Functional outcomes were equivocal where the control arm demonstrated faster timed 10 m walk test and time up-and-go test and intervention arm patients demonstrating a higher 30s chair stand test. None of the results reached statistical significance. Table 7 summarises the outcome measures.

**Table 7:** 12-weeks Outcome Measures

Outcome Measure	Improvement in Control (Median, IQR)	Improvement in Intervention (Median, IQR)	*p*-value
KOOS score			
KOOS_4_	9.46 (30.28)	21.38 (21.38)	0.34
KOOS symptoms/stiffness	5.36 (43.75)	28.57 (39.29)	0.28
KOOS pain	13.89 (33.34)	25.00 (8.33)	0.39
KOOS function (daily living)	10.30 (34.19)	22.88 (13.24)	0.63
KOOS quality of life	12.50 (32.81)	25.00 (25.00)	0.41
Quality of Life			
EQ-5D Index	0.46 (0.67)	0.34 (0.31)	0.56
EQ-5D VAS	10.00 (30.00)	15.00 (20.00)	0.47
Psychology			
PEG	−1.50 (6.00)	−2.33 (2.33)	0.89
PHQ-4	0.00 (1.75)	0.00 (4.00)	0.18
Weight	1.80 (1.97)	−0.30 (1.90)	0.59
Functional Assessment			
Timed 10 m walked test (sec)	0.44 (1.63)	0.29 (1.30)	0.45
Time up-and-go test (sec)	1.23 (4.31)	0.40 (1.61)	0.20
30s chair stand test (repetitions)	2.00 (1.50)	3.00 (2.00)	0.23
4 stairs climb test (sec)	0.62 (8.54)	1.59 (3.06)	0.42

For all the patients with BMI > 23.5, all the patients in the intervention arm (*n* = 3) demonstrated positive change in their dietary habits based on the modified FFQ compared to the control arm (*n* = 7) where only 57.1% of patients demonstrated a positive change in their dietary habits after 12-weeks.

One patient in the intervention arm suffered adverse events. The patient developed concurrent back pain during the course of the program. It was ascertained that the patient had long standing low back pain which was exacerbated during the intervention. Assessment by an independent physiotherapist deemed that the exercises prescribed were unlikely to cause the exacerbation. The back exacerbation was treated successfully with physiotherapy and analgesia. One patient in the control arm who deteriorated was subsequently diagnosed with spontaneous osteonecrosis of the knee and underwent knee arthroplasty.

### Progression criteria results

All domains except patient recruitment met progression criteria to proceed with the RCT (green light) based on the evaluation by the study team. Over a 3-month period, only 20 patients were recruited. Based on the pre-determined progression criteria, an allowance of 6 months was permissible to determine if the target of 30 patients was achievable distinguishing between the amber and red light. While recruitment of 30 patients over a 6-month period was potentially possible for this pilot study, such a recruitment rate was not feasible for the main trial in view of grant funding time restrictions and logistical considerations for the main trial that were not known during the conceptualization of the pre-determined progression criteria. A decision was made to classify this as a red light prompting significant changes to be made to achieve a green light before the main trial could proceed. Table 8 summarizes the feasibility for full RCT based on the progression criteria.

**Table 8:** Progression Criteria

Domain	Results	Readiness for Progression
Patient Recruitment	20 patients	Do not proceed with RCT unless problem can be solved
Patient Retention	85%	Proceed with RCT
Intervention Completion	80%	Proceed with RCT
Outcome measure acceptance	100%	Proceed with RCT
Improvement in function and/or quality of life (Intervention arm)	88.8%	Proceed with RCT
Serious Adverse events	0	Proceed with RCT

### Process evaluation

A total of 8 patients in the intervention arm were interviewed as part of the process evaluation. Two patients in the intervention arm declined to be interviewed.

The first focus was on intervention optimization. Several themes were identified. Firstly, for the exercise component, all the patients felt that it was beneficial. Three patients felt the number of sessions could be increased with an additional 2 sessions for greater benefit. Learning different exercises techniques and how to adapt them based on individual fitness and needs was a key benefit that patients reported. While patients recognized that it was beneficial to exercise, there was a realization that they were unlikely to return to normal function. This point was emphasized during the education and psychology sessions where an acceptance of the irreversible effects of aging was important while at the same time, recognizing how exercise can help patients cope better with these changes.

*“It’s good to learn about the different exercises techniques and how to improvise them” (P002).*

*“Knee condition seems to improve but don’t think it will go back to normal” (P019).*

Secondly, for nutrition and dietetic components, patients felt that while most of the dietary information was not new to them, the emphasis on “mindful eating” was particularly useful where patients were taught to actively monitor their dietary intake instead of taking a passive stance.

Thirdly, for the psychology sessions, there was an initial reluctance due to social stigma that psychology intervention was associated with mental illness such as depression. However, the patients felt that it was very beneficial upon completion of the program. Enhancing self-management was a common theme that many patients felt would help them maintain their improvement.

*“It serves as a reminder to direct our mindsets to a positive direction” (P013).*

Fourthly, in terms of general feedback received, there was very positive feedback for an intact group concept where patients were kept together throughout the 12-week program instead of having patients constantly moving in and out of the program. They felt that the community setting was welcoming and many of the patients looked forward to the sessions. Some patients shared information about how they wanted to keep in touch even after the program concluded. Many patients expressed some form of positive peer pressure from fellow patients.

*“Happy to see the same faces….. making friends” (P012).*

*“You feel motivated to be even better when you see others improved over time” (P002).*

*“Everyone is very caring and accommodating. Feels warm coming to the program” (P013).*

The second focus of the process evaluation and interview was the feasibility of a larger trial. All the patients in participating in the study did not express any regret participating however reporting significant difficulty understanding certain psychological outcome measures (AAQ-II [47], CPAQ-8 [48], GIC [49]) and thus the majority of these outcome measures were not able to be completed by the patients. Several patients expressed that they would recommend their friends suffering from similar conditions to participate in the program.

## Discussion

The CONNACT model of care is a complex intervention consisting of several different components interacting with each other. The Medical Research Council guidance on developing and evaluating complex interventions recommends a feasibility and piloting phase at the start prior to a full study [59]. The primary aim of the pilot study was to determine the feasibility of a full RCT through pre-defined progression criteria. The secondary aim was to optimize the intervention and study design through a process evaluation in preparation for a full RCT. Results from the pilot affirmed the feasibility of the study to progress to a full RCT. Secondly, results from the process evaluation through the interviews informed trial design methodology and intervention optimization.

### Feasibility of a full RCT

Based on the proposed progression criteria, all the areas of the pilot were ready to proceed with a full RCT except the recruitment aspect where only 20 patients were recruited for the study. Avery et al. proposed solutions in the event of insufficient recruitment including exploring screening logs to determine if insufficient participants were approached, passed eligibility criteria or agreed to randomization. After a throughout review of our screening logs and recruitment process, the primary reason identified was insufficient patients were screened and directed to the recruitment clinic during the recruitment period. Based on the recruitment rate from the pilot, a wider net for screening of all referrals and additional recruitment clinics are planned during subsequent recruitment cycles for the sample size calculations to be fulfilled within the duration of the study.

The pilot study results suggested that patients who underwent the intervention were more likely to have a better knee function score, better quality of life, have less anxiety and depression, lose weight and exhibit a positive dietary change compared to control arm patients. Although none of the results reached statistical significance potentially owing to low patient numbers, these results are promising.

Compared to taking reference values from other studies, results from this pilot study including the mean values and standard deviations of the primary outcome, KOOS_4_ will be used for a more representative power analysis to calculate the required sample size for the main RCT.

### Proposed changes to intervention

Results from the qualitative study informed changes to the intervention protocol that could potentially enhance its effectiveness. The number of exercise sessions will be increased from 6 to 8 sessions in line with international programs which can utilize up to 24 sessions over a 12 week period [19]. The dietetic syllabus will be modified to focus more on “mindful eating” and practical examples on how to make a sustainable diet change compared to simply giving patients a dietetic lecture on the relative health benefits of different food groups. The group-based intervention format will be retained. Group-based interventions have been shown to be more effective compared to individual interventions [36]. A flexible post-intervention program will be developed for patients who would like continue to exercise together in a group and social network platforms e.g. Whatsapp group chats or Facebook groups will also be utilized to facilitate patient group interaction throughout and post-intervention. Keeping a group-based program does pose logistical challenges. In order to overcome this, recruitment for the main trial will be done in cycles to group patients into classes and retain this key element.

In line with the overall thrust of enhancing patient self-management, patient activation is a key concept that will be included as part of the intervention. Patient activation is defined as an individual’s propensity to engage in adaptive health behaviour that may lead to improved outcomes. Activation levels is measured by the Patient Activated Measure (PAM) [60], a validated questionnaire that looks at knowledge, skills and confidence in managing health. There has been increasing evidence in the literature that high PAM scores have been associated with more satisfaction with healthcare services, better self-management behaviour and improved health outcome [61, 62]. In addition to PAM being added in as an outcome measure, PAM levels of less than 3 will also serve as an independent eligibility criterion for psychological intervention. Several key areas has been identified when including activation as part of any intervention, including physician-patient relationships, self-management, facilitating behaviour change and tailoring interventions according to activation levels [63]. Based on these principles, a greater emphasis on patient activation will also be included into the psychological intervention.

A recent Cochrane review on exercise interventions and patient beliefs for people with hip or knee OA revealed that many patients are confused about the cause of their pain and are unsure about what steps they should take to manage their pain generally resulting in activity avoidance for fear of causing harm [64]. Evidence has shown the potential that proper education and behaviour modification has in producing long lasting sustainable positive effects in OA programs [65]. Proposed topics for the education sessions are based on areas of patients’ knowledge deficiencies identified by the Cochrane review. These topics include the following.

1. Pathophysiology behind OA
2. Flare management – what causes flares and how to deal with them
3. Treatment options and their relative effectiveness, pros and cons

“Expert patients” is a relatively novel concept where patients who have successfully completed the program are invited to share their experiences with the incoming batch of participants. Expert patients have previously been included in similar programs [28]. “Expert patients” will be incorporated in the main trial intervention as part of the educational component as volunteers. They will be recruited from previous cycles of patients who have successfully completed and benefited from the intervention.

### Proposed changes to methodology

There were several proposed modifications to the choice of outcome measures both based on the process evaluation and additional literature review after review of the pilot study results. Firstly, some of the psychological questionnaires (AAQ-II [47], CPAQ-8 [48], GIC [49]) were removed as all the patients had difficulty understanding resulting in completion difficulty. Other outcome measures such as the PAM, Global Perceived Effect (GPE) [49], Patient Acceptable Symptom State (PASS) and treatment failure [66] which are sensitive but simple questionnaires will be included for the subsequent RCT in place of the excluded questionnaires as these outcome measures have previously been used in musculoskeletal conditions of the knee [67]. The overall respondent burden would be reduced.

Compliance to exercises is a key outcome measure and a potential confounder when interpreting trial results that was not measured in the initial pilot. In the current literature, there is a lack of validated questionnaire that reliably measures exercises compliance or adherence [68]. Compliance can be assessed in 2 different ways, either through patient reported measures or clinician assessment. For the main trial, a comprehensive assessment for compliance was deemed to be crucial due to nature of intervention where patient participation played a critical role. For patient reported outcomes, a simple questionnaire will be developed focusing on exercises compliance and reasons for non-compliance that will be administered to the intervention arm patients at 3 months, 6 months and 12 months. For clinician assessment, the Sports Injury Rehabilitation Adherence Scale (SIRAS) [69] is a validated tool for compliance assessment.

### Strengths

This paper highlights a comprehensive approach of a feasibility study using a pilot randomized trial prior to an RCT for a complex intervention. Firstly, progression criteria based on established guidelines was developed prior to the conduct of the pilot study as an objective benchmark to decide if a full RCT was feasible at that point in time. These proposed progression criteria can be adopted and evaluated by other pilot studies looking at similar interventions for musculoskeletal conditions.

Secondly, a process evaluation guided by the MRC guidelines [58] was embedded within the pilot study through qualitative methods focusing on intervention optimization and feasibility. This allowed for informed modifications to be made to both the intervention and study methodology to give the subsequent RCT every chance of success.

### Limitation

We were only able to recruit 20 patients instead of the targeted sample size of 30 patients. While sample sizes for pilot studies are less critical in ensuring adequate power, this highlighted a key area moving forward for our main RCT. Recruitment was one of the key elements described in the progression criteria. Our patient recruitment strategy was critically evaluated and key changes will be made for the main trial.

There were significant differences between the control and intervention population group in terms of gender distribution and mean age. The mean age in the control arm was significant younger and had more males compared to the intervention arm. In general, there were significantly more females compared to males in both the intervention and control arm. This could be a result of the small sample size. This issue will likely be addressed during the main RCT where a much large sample size will be targeted and use of stratified randomization to control for gender.

## Conclusion

This pilot has demonstrated the feasibility of a full RCT investigating the potential effectiveness of the CONNACT model of care for knee OA using pre-defined progression criteria and process evaluation. Results from the qualitative study were used to modify and improve the intervention content, delivery model and study design for a large effectiveness-implementation hybrid RCT that is currently underway. This main trial includes 1-year follow-up, economic evaluation and process evaluation using the MRC guidelines [58], the RE-AIM (Reach, Effectiveness, Adoption, Implementation, Maintenance) implementation and evaluation framework [7] and the Global Alliance for MSK Health (GMUSC) framework [26] to guide large scale implementation.

## Data Availability

The datasets used and/or analysed during the current study are available from the corresponding author on reasonable request.

## Abbreviations

CONNACT: Collaborative Model of Care between Orthopaedics and Allied Health Professionals Trial
MSK: Musculoskeletal
OA: Osteoarthritis
RCT: Randomized Controlled Trial
NICE: National Institute of Health and Care Excellence
ACSM: American College of Sports Medicine
NEMEX: Neuromuscular Exercise
OARSI: Osteoarthritis Research Society International
KOOS: Knee Injury and Osteoarthritis Outcome Score
EQ-5D-5L: EuroQol 5 dimensions 5 level
BMI: Body Mass Index
PHQ-4: Patient Health Questionnaire 4
PEG: Pain, Enjoyment, General Activity Scale
ACT: Acceptance and Commitment Therapy
AAQ-II: Acceptance and Action Questionnaire 2
CPAQ-8: Chronic Pain Acceptance Questionnaire 8
GIC: Global Impression of Change
FFQ: Food Frequency Questionnaire
ITT: Intention-to-treat PAM Patient Activation Measure
GPE: Global Perceived Effect
PASS: Patient Acceptable Symptom State
SIRAS: Sports Injury Rehabilitation Adherence Scale
MoC: Model of Care
IQR: Interquartile range
SD: Standard Deviation
